# Soybean Development: The Impact of a Decade of Agricultural Change on Urban and Economic Growth in Mato Grosso, Brazil

**DOI:** 10.1371/journal.pone.0122510

**Published:** 2015-04-28

**Authors:** Peter Richards, Heitor Pellegrina, Leah VanWey, Stephanie Spera

**Affiliations:** 1 Population Studies and Training Center, Institute for the Study of Environment and Society, Brown University, Providence, Rhode Island, United States of America; 2 Department of Economics, Institute for the Study of Environment and Society, Brown University, Providence, Rhode Island, United States of America; 3 Department of Sociology, Population Studies and Training Center, Institute for the Study of Environment and Society, Brown University, Providence, Rhode Island, United States of America; 4 Department of Geological Sciences, Institute for the Study of Environment and Society, Brown University, Providence, Rhode Island, United States of America; College of Agricultural Sciences, UNITED STATES

## Abstract

In this research we consider the impact of export-driven, soybean agriculture in Mato Grosso on regional economic growth. Here we argue that the soybean sector has served as a motor to the state’s economy by increasing the demand for services, housing, and goods, and by providing a source of investment capital to the non-agricultural sector. Specifically, we show that each square kilometer of soybean production supports 2.5 formal sector jobs outside of agriculture, and the equivalent of approximately 150,000US in annual, non-agricultural GDP. We also show that annual gains in non-agricultural employment and GDP are closely tied to soybean profitability, and thus vary from year to year. However, while this article highlights the potential of the agricultural sector as a driver of regional economic growth, it also acknowledges that this growth has been sustained by profits determined by externally set prices and the rate of exchange, and that future growth trajectories will be susceptible to potential currency of market shocks. We also show that while Mato Grosso’s economic growth has come at a significant cost to the environment, value added by the agriculture sector, directly and indirectly, has surpassed the value of the CO_2_-e emitted through land clearings.

## Introduction

Between 2000 and 2005 Brazil’s soybean area expanded from 13.6 to 23.4 million hectares. Mato Grosso State, on its own, accounted for nearly four million hectares of this expansion [1]. The environmental costs of this growth have been carefully documented. Soybean production in Mato Grosso has been widely tied to deforestation, directly, via the conversion of forest areas to cropland, and indirectly, through the sector’s impact on regional land markets and investment decisions [2–5]. In this article we consider the impact of soybean production not on the regional environment, but on regional economic development. Specifically, our objective in this research is to estimate urban socioeconomic change as a function of local agricultural production. Here we show that over the past decade, in Mato Grosso, soybean agriculture has led to rapid growth in formal sector employment outside of agriculture (services, commerce, construction and public administration, education and health), in non-agricultural GDP, in urban population, and in nighttime light emissions, a proxy measure for economic activity. We also show that this growth is closely tied to returns to soybean production. Consequently, we argue that while export oriented agriculture may pose a viable channel for broader economic growth in non-agricultural sectors, this growth will remain dependent on exogenously determined input costs and market prices.

This research directly engages with ongoing discussions over the potential of agricultural systems built on exports to serve as drivers of local economic development [6–11]. In Mato Grosso, or in Brazil more broadly, evidence increasingly suggests that soybean agriculture, which is largely producing for international markets, has had an important impact on regional development. Most notably, previous research has suggested that while soybean producing areas exhibit increasing levels of inequality, they also exhibit higher median incomes, higher human development indices, lower poverty rates, and better schools [12–14]. Our work builds on these past studies by not only recognizing and measuring the impact of soybean agriculture on a series of socioeconomic indicators, but by examining the effect of soybean production on employment and non-agricultural economic activity.

To conduct our analysis we draw on methods from both econometrics and spatial analysis, and from a spatial dataset of biophysical and social indicators. Specifically, we focus on the influence of agriculture on changes in (1) nighttime light emissions (a measure of urban economic activity); (2) urban population; (3) non-agricultural GDP; and (4) non-agricultural employment. Our results suggest that not only is soybean production leading to positive gains in these socioeconomic variables, but that the year by year magnitude of change closely tracks regional returns to production. While we show that commercial agricultural systems can have an impact on non-agricultural sectors, we nevertheless caution that institutional and natural conditions for agriculture in Mato Grosso may have amplified the economic impact. However, in an appropriate context, we suggest that policymakers seeking to promote economic development may wish to focus on developing high-return, market-oriented crops, in addition to supporting widely distributed improvements in productivity among small farmers.

## Background

**Fig 1:**
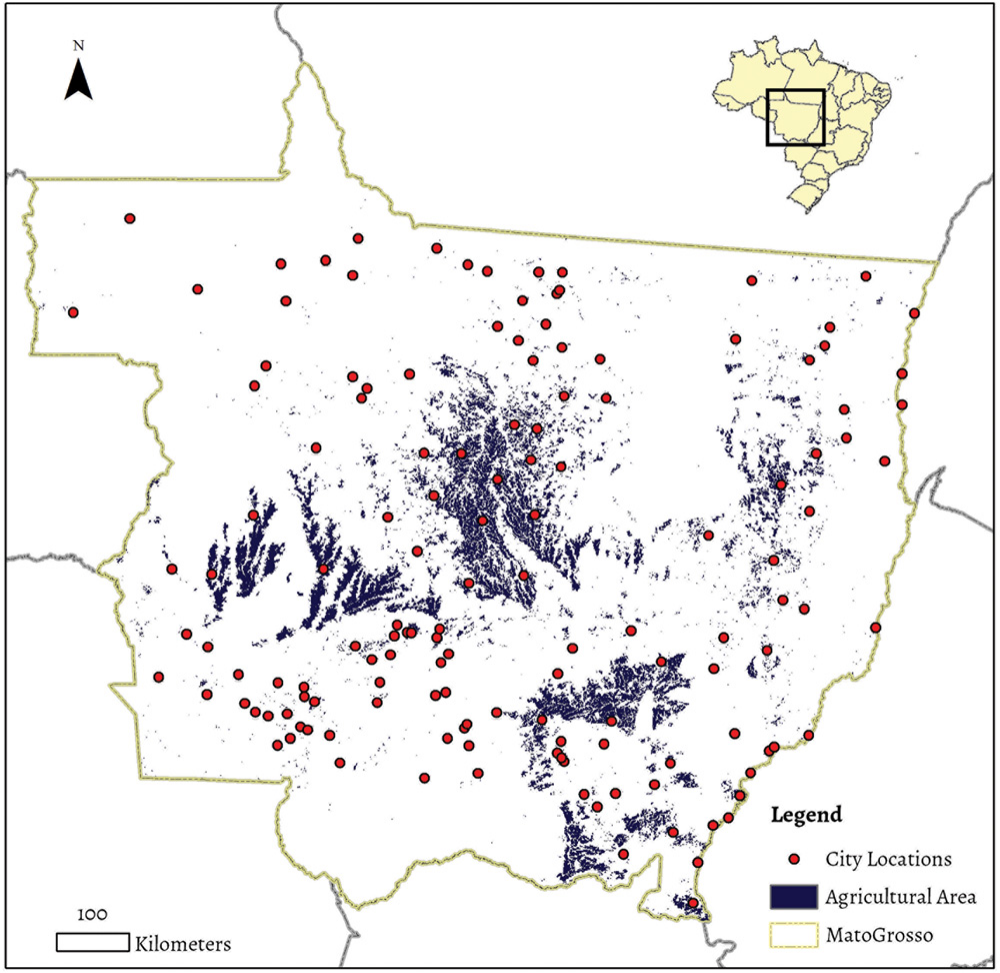
Mato Grosso’s agricultural regions and urban areas. Agriculture is concentrated in the central-north, west, southeast, and eastern areas of the state.

Research on agriculture as a driver of economic development has widely recognized cross-sectoral linkages between agriculture and non-agricultural economic sectors [8,9,15–17]. In this article we follow work by Johnston and Mellor, which suggests that agriculture generates economic growth in non-agricultural sectors by increasing the demand for local consumption goods, and by increasing the local supply of investment capital. In this sense, a robust agricultural sector can provide a source of investment capital to the non-agricultural sectors [8,15]; lead to the development of upstream and downstream service sectors, and even light manufacturing; and increase the demand for goods and services such as housing, education, entertainment, and purchased food and material goods. This perspective follows directly from arguments suggesting that agricultural development may serve as an agent for poverty reduction [6,8]. We argue that in Mato Grosso, Brazil (Fig 1), agriculture has led to economic growth through three potential channels: (a) the development of upstream support sectors and downstream linkages to the agriculture sector; (b) increased demand for non-tradable services and consumption goods (provided or sold by local labor); and (c) the flow of profits from agriculture into non-agricultural investments.

**Fig 2:**
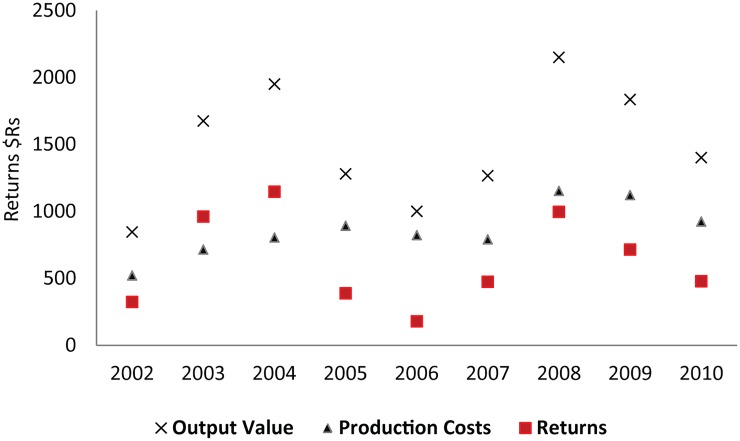
Production costs and profits in $Rs per hectare of soybean production, 2002–2010. Profits were low in the middle of the decade, and high during the period 2002–2004 and in 2008. Assumes productivity of 3,000kg/ha. Production costs include seed, labor, diesel and chemical inputs for Primavera do Leste, MT [18]. Prices based on 60kgs of soybeans on or near to March 1^st^, in Primavera do Leste, MT [21] Returns = output value–production costs.

In Mato Grosso, the soybean sector has an outsize role in the state’s economy. In 2010 production costs per hectare in Mato Grosso, including seed, chemical inputs, and labor, amounted to approximately 535$US per hectare (As we show in Fig 2, total costs per hectare were 923.56$Rs. We converted to dollars by the 2008 rate of exchange, which we estimated at 0.58) [18]. Given that in 2010 the state’s farmers planted 6.2 million hectares [19], statewide, planting costs for that year alone amounted to more than 3 billion $US. Not surprisingly, a complex support sector has evolved to finance, distribute, advise on, and sell agricultural inputs in the region [20]. Agro-consulting companies now help farmers decide what technology to use, vendors sell the expensive harvesting, planting and spraying machines, and fertilizer and grain companies compete to sell inputs and finance farmers’ crops. These businesses bring new employment to the region, often to the urban areas that service the region’s agricultural sector.

Downstream linkages are also emerging in the state. Soybean crushing facilities, biodiesel and ethanol plants, and poultry, swine, fish, and confined beef operations, each of which rely on locally-produced corn and soybeans, are now located in the state [22]. For the soybeans that head out of the region, the crop must also be purchased by a grain company, temporarily stored, and then transported to a distant port. Downstream processing linkages, as with the legions of service providers located upstream in the production process, are often located in urban areas, and have contributed greatly to regional urban growth.

We also theorize that agriculture drives urban growth through demand for consumption goods, and by increasing the supply of locally available investment capital. In Mato Grosso the agriculture sector serves as a key generator of capital, particularly in years of high returns. For example, in 2008, Mato Grosso harvested 17.8 million tons of soybeans from 6.2 million hectares of cropland (approximately 3.15 t/ ha). Given that at harvest time that year (early March), one ton of soybeans was worth approximately 716$Rs (or about 415$US [21]), the state’s soybean harvest was worth more than 12$Rs billion (7$US billion). Even after accounting for production costs (in 2008, 1,153$RS/ha), that year, state-wide, farmers grossed approximately 3.6 billion $US in profits. In 2008, for a single farmer planting 1,000ha (a size typical in the northern regions of the state) the one harvest might generate a profit of more than half a million dollars. And for landowners renting their land to soybean farmers, at a rate of approximately ten 60kg/bags per hectare (a standard rate in the more heavily farmed areas), renters would gross approximately 250$US per hectare. For a landowner renting 1,000 hectares of farmland, the rental value alone could amount to 250,000$US. In this context, soybean farming can generate impressive returns in Mato Grosso. A key question, however, is how these profits are then spent.

We theorize that much of the profits generated in agriculture are consumed or invested locally. In Mato Grosso, and in Brazil more broadly, traditionally, many investors have used land as a safe haven for storing capital (e.g., rather than banks or bonds). Today, as land is increasingly expensive, many farmers will also invest in sectors with downstream or upstream linkages to farming (agro-consulting or swine production, or silage facilities), or in the non-agricultural sector: in fuel stations, restaurants, or urban real estate. Some farmers might invest surplus capital in family members’ non-agricultural business, or to purchase urban properties. Consequently, farming can provide a key source of investment capital to local urban areas, particularly where farmers live locally.

Following these observations, we expect that all else being equal, cities surrounded by a larger base of agriculture should exhibit higher economic and demographic indicators (from GDP to employment, nighttime light emissions and urban population) than peer cities surrounded by less intensive land uses. We also expect that these cities will grow faster when profits are highest. During years of high returns the demand for consumption goods and merchandise (e.g., housing, trucks, cars, clothing, and entertainment) will increase, and farmers will have more surplus capital to invest, either in expanding agricultural production or intensifying on existing farmlands, or in non-agricultural investments. Consequently, we argue that not only are soybeans driving economic growth, but that their ability to do so is closely tied to production returns.

## Materials and Methods

**Fig 3:**
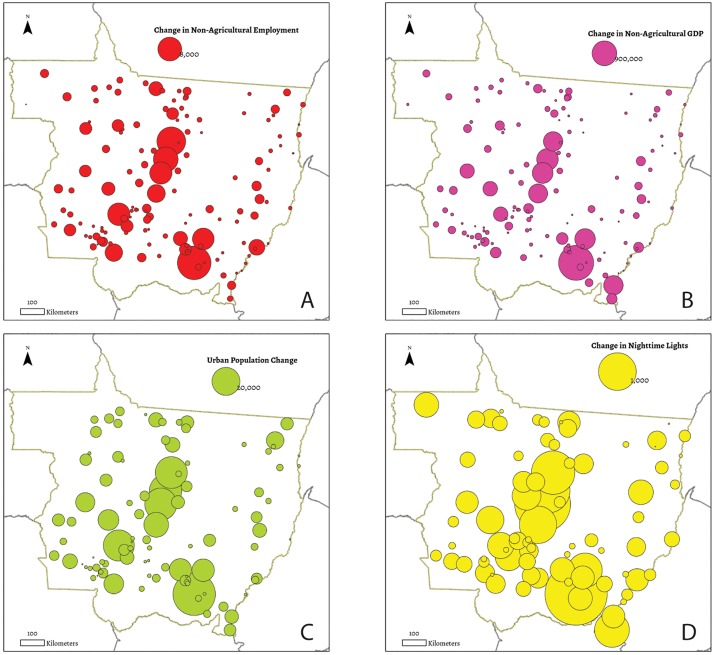
Socioeconomic change in Mato Grosso. Change in (a) non-agricultural employment,(b) non-agricultural GDP; (c) urban population in Mato Grosso between 2001 (2000 for urban population) and 2010, and (d) nighttime lights. Capital regions are excluded. Largest changes are concentrated in cities in agricultural regions.

Our objective in this research is to estimate urban socioeconomic change as a function of urban areas’ surrounding agricultural production. We focus on four principal socioeconomic variables as dependent variables: (a) formal sector employment in service, the public sector, health, education, construction, or commercial sectors [23]; (b) non-agricultural GDP (in 1,000s of $Rs, deflated to 2001 values, see Table 1 in S1 Manuscript) [24]; (c) census records of urban population [25,26], and (d) urban nightlights, which have been regularly used as a proxy for urban economic or population growth [27–31];. We show changes in each variable over the last decade in Fig 3a–3d. We note that we omit Mato Grosso’s capital region from our analysis (Cuiabá and Várzea Grande), given that these areas are, by political design, less reliant on absorbing capital generated from their immediate surroundings.

**Fig 4:**
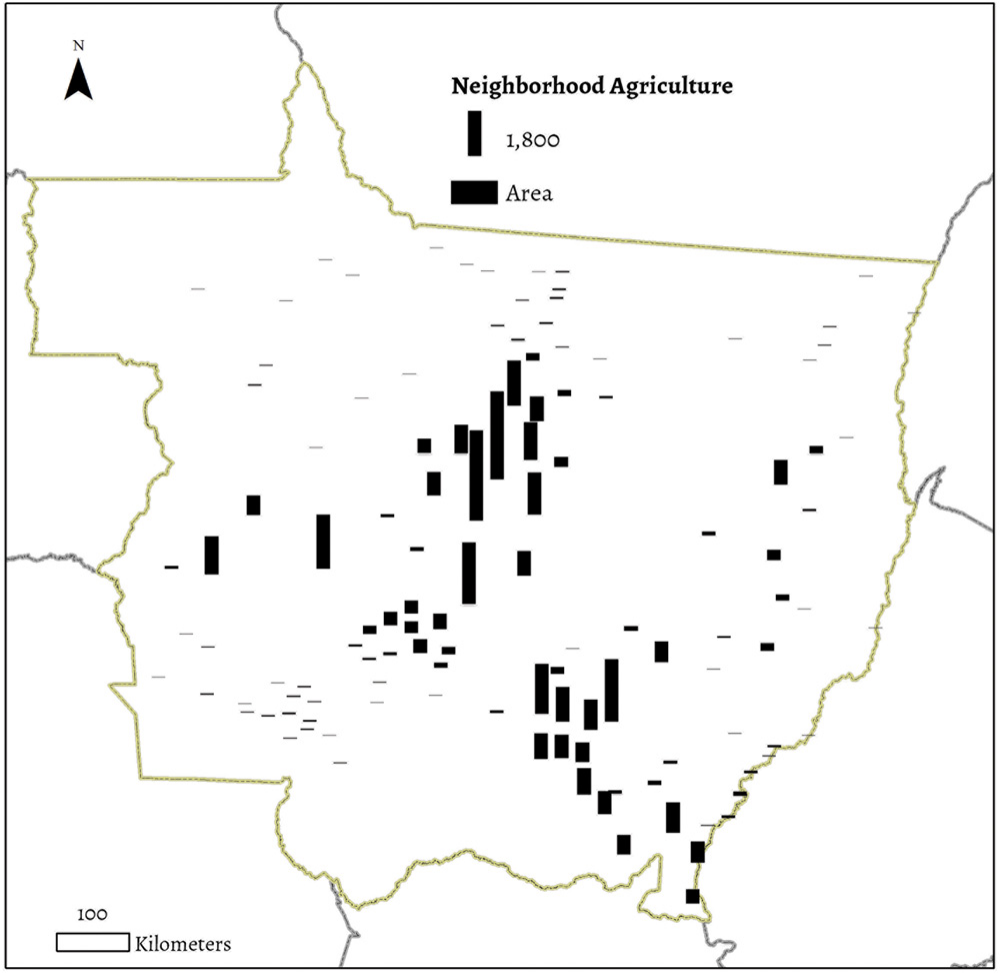
Total agriculture (km²) by city neighborhood, 2010.

The explanatory variable of key interest to our work is the area of soybean agriculture in the vicinity of each city in Mato Grosso. To calculate soybean area in each city’s neighborhood we first estimated travel times between each city and its surrounding region using the state’s road network (see *SI* for a complete description of methods). We then set a neighborhood threshold at sixty minutes, and classified areas accessible within sixty minutes driving as part of a city’s neighborhood. Finally, we calculated total agricultural area in each city’s neighborhood, using land use classifications based on MODIS satellite data [32]. Neighborhood agriculture thus refers to the total area of agriculture, in this case the total square kilometers of soybean production, located within sixty minutes driving of each urban area (Fig 4).

We focus on total agricultural area rather than change in agricultural area as our key explanatory variable, as we theorize that the principal impact of agriculture lies in the ability of the agricultural sector to not only increase demand for upstream and downstream linkages, but also to generate investment capital and new demand for consumption goods. We do not account for other agricultural systems or pasture production directly in this analysis. However, we do include a control variable for total area of cleared land in each city’s neighborhood [33]. In Mato Grosso, the vast majority of cleared land not used for soybean agriculture is used as pasture.

We included a set of control variables in our specifications to control for factors that might be correlated with both agricultural production and the generation of jobs in the urban centers: (a) total distance between each city and São Paulo, Brazil’s economic center, which also happens to be located in close proximity to the port of Santos, which is Brazil’s principal soybean export port; (b) the distance between each city and the nearest historical federal highway and major river; and (c) the most common soil type and texture in each city’s surrounding neighborhood (see Table 1 for a full list of variables; mean descriptive statistics are included in the SI as Tables S2–S3). The results are largely robust to the inclusion of these controls variables.

**Table 1:** List of key variables.

Dependent Variables	Explanatory Variables
Formal sector, non-agricultural employment	Agriculture^N^
Non-agricultural GDP	Km to São Paulo
Nighttime light emissions	Km to nearest principal road
Urban Population	Km to nearest main river
	Neighborhood elevation^N^
	Slope^N^
	Soil type^N^
	Soil texture^N^
	Non-Forest^N^

^N^ Denotes neighborhood variable

We test four sets of specifications. In specification sets one and two we regress total economic activity (e.g., total, non-agricultural, formal sector employment and GDP) on total neighborhood agricultural production. Specification set one uses a fixed effects specification, with city (group) and year effects. These specifications produce a single estimate of non-agricultural GDP and formal sector employment supported by agriculture over the course of the past decade. In specification set two we again regress total formal sector employment and GDP on neighborhood agriculture. However, in these specifications we slice the panel into one year intervals and replace the group effects with a vector of local control variables. These specifications produce an estimate of agricultural spillover effects for each year over our period of analysis. We expect that the spillover effects will have decreased over the past decade, as local processing capacities (e.g., crushing facilities, poultry production, biodiesel and ethanol plants) and agricultural services have increased.

In specification sets three and four we use *changes* in each socioeconomic indicator as our dependent variable. These specifications follow from the argument that agriculture will drive local growth by increasing local demand for consumption goods and services (e.g., housing, schools, entertainment), and as a source of investment capital. We expect that the impact of agriculture on urban change will follow agricultural returns. To test this hypothesis, in specification set three, we regress change in non-agricultural, formal sector employment and GDP on neighborhood agriculture, in one year intervals. Finally, to estimate the effect of agriculture on urban socioeconomic change over the full decade, we test a fourth set of specifications. Here we regress change in each of socioeconomic variable over the full time frame of our analysis (2001–2010 for employment, non-agricultural GDP data, and nighttime lights, and 2000–2010 for urban populations) on a vector of control variables and neighborhood agriculture.

### A note on intersectoral feedback effects

Past research on agriculture as a driver of economic development has shown that disentangling the impact of the agriculture sector on broader economic growth from reverse feedbacks can be a difficult challenge [8]. For while agriculture may serve as a driver of economic growth, research also shows that in certain cases the non-agricultural sector may drive agricultural intensification or expansion. In theory this could occur through several channels. For example, growing and wealthier urban manufacturing and service sectors might increase the demand for local agricultural goods, and lead to an increase in agricultural returns and growth in the local agriculture sector. Alternatively, higher wages in urban areas could draw labor out of rural areas and incentive productivity advances. If urban growth is driving agricultural change in Mato Grosso, then our estimations would be biased.

In Mato Grosso, we argue that local urban growth is not a driving factor behind the region’s growth in soybean production. First, in this research we focus exclusively on Mato Grosso’s soybean sector (cotton and corn which are often double cropped with soybeans are also included in our estimates of agriculture area), which is largely produced for export markets. Because it is not local diets that are driving the changes in demand for soybeans, but rather changes in consumption that are occurring in potentially very distant locations (e.g., in São Paulo of East Asia), production decisions are not likely to be significantly influenced by changing diets in local cities. Second, by analyzing growth at the municipal level we are able to use the natural spatial heterogeneity of agricultural production within the state as a differentiating factor underlying local growth. This allows us to examine the impact of local soybean production in a region that has a small manufacturing sector, much of which is concentrated in the state capital area that is already excluded from our analysis. Third, many of the soybean regions of Mato Grosso were, until recently, frontier areas. To support both rural and urban growth, new labor must be brought in through in-migration. Many of the rural soybean regions exhibit positive net-migration, which suggests that these regions are not merely shedding labor to urban areas. Nevertheless, to provide further robustness to our results and to deal directly with concerns about simultaneity bias in our results, we compare the estimates obtained in our specifications against those obtained using an instrument for agricultural area that is arguably exogenous to urban growth. We include these results in the SI.

## Results

### Specification Set 1: Annual Estimations, Fixed Effects

Our first set of specifications uses the full panel extent (2001 to 2010) of the non-agricultural employment and GDP data in a fixed effects model. Here we regress socioeconomic indicators on neighborhood agricultural production and neighborhood non-forest area (the only control variable that, like agriculture, will expand with time) during the previous year, along with city and year effects.

In each specification we estimate positive and significant coefficients associated with neighborhood agriculture. For example, we estimate that each square kilometer of agriculture has a spillover effect of 2.18 new, formal sector, non-agricultural jobs and an additional 181,000$Rs in non-agricultural GDP (Table 2). In 2010 values, this is equivalent to a spillover effect of slightly more than 300,000$Rs, or about 168,000$US (average $Rs:$US exchange rate in 2010 was 0.56).

**Table 2:** Fixed effects estimation results, specification set 1.

Specification Set 1: 2010–2001, Panel data using fixed effects. N = 1250; 139 Groups		
Specification:	Employment	GDP^+^
Agriculture^N^	2.18** (0.86)	181** (58.6)
Non-Forest^N^	0.35 (0.58)	-16.8 (38.4)
Group Effects	Yes	Yes
Year Effects	Yes	Yes
(within) R²	0.22	0.19
Panel Years	2010–2001	2010–2001
N	1251	1245

**: p <. 01

^N^ denotes neighborhood variable, e.g., total agriculture and non-forest within a city’s sixty minute neighborhood.

^+^ In 1,000s of $Rs, deflated to 2001 values

### Specification Set 2: Annual Estimations, OLS

As with specification set one, the second set of specifications also regresses total non-agricultural GDP and formal sector employment on neighborhood agriculture. However, here we split the dataset to evaluate the effect of agriculture in one year intervals. In this second set of specifications, we have only one observation per county in each regression and cannotinclude fixed effects to reduce the role of unobserved omitted variables that are constant overtime. To deal with this limitation, we replace the group effects with local control variables (see Table 1). The full results from these specifications are included in Tables S4–S5; however, we focus on the coefficients associated with agricultural area on change in the dependent variable.

**Fig 5:**
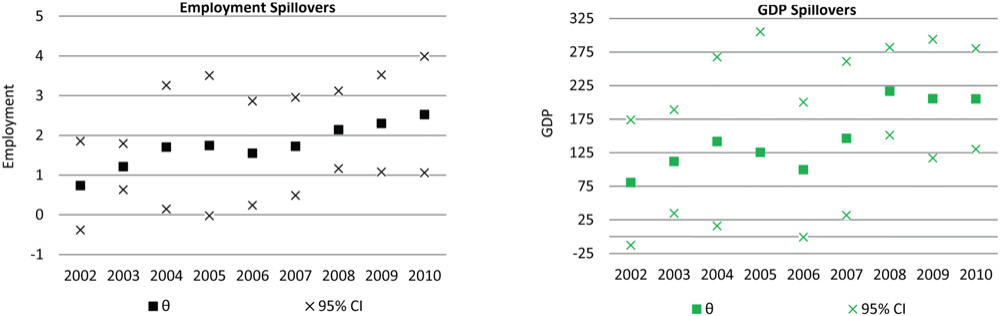
Total non-agricultural employment (5a) and GDP (5b) attributed per 1km² of soybean production, 2002–2010.

In Fig 5a and 5b we plot the coefficients associated with neighborhood agriculture (shown as θ) on non-agricultural employment and GDP. For both variables, θ increases steadily. For employment, θ increases from 0.73 to 2.5 over the course of the time span, with only a slight decrease during the middle years of the decade, when returns were low. This effect is more pronounced in our estimates of θ in our specifications of non-agricultural GDP. For the first year of this specification set, namely 2002, we estimate that each 1km² of agriculture generated an additional 80,000$Rs in non-agricultural GDP. However, this increased to 215,000$Rs by 2008, before stabilizing at 205,000$Rs for 2009 and 2010 (values in 2001 levels). In 2010 values, this equates to approximately 375,000$Rs, or about 210,000$US in spillover effects, per square kilometer of agriculture.

Our estimates of θ from this second set of specifications suggest that the impact of soybean agriculture on the non-agricultural sectors in northern Mato Grosso has grown steadily over the past decade. Arguably, this pattern reflects the increased labor and capital invested in the upstream and downstream support sectors to agriculture in the region. Alternatively, it may reflect increasing productivity in the agricultural sector, or the expansion of double cropping planting strategies in the region after 2006.

### Specification Set 3: Annual Change, OLS

In our third set we estimate the influence of agriculture on annual *change* in each socioeconomic indicator. Here we regress annual change in non-agricultural GDP and formal sector employment on total neighborhood agriculture and the vector of local control variables. This will allow us to compare our estimates of the impact of agriculture with the profitability of soybeans in each different year. Note that our measure of profitability is completely independent from our estimation procedure. Therefore, without a theoretical link between these two measures (the impact of agriculture and the measure of profitability), there is no reason to expect a mechanical correlation between them. The full results from these specifications are included in the SI as Tables S6–S7; however, as with the results of our second set of specifications, we focus on the coefficients associated with agricultural area, θ.

**Fig 6:**
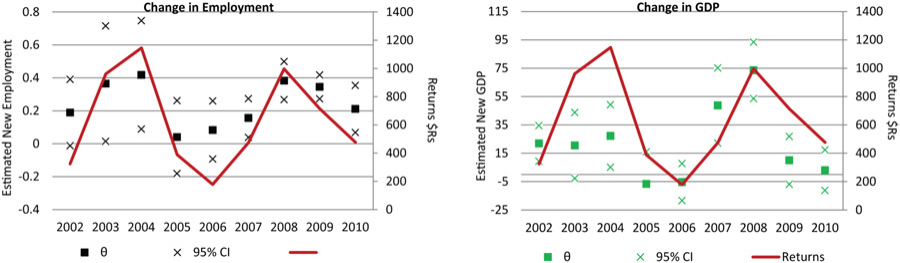
Estimated change in (6a) formal sector, non-agricultural employment and (6b) non-agricultural GDP. GDP shown in 2001 $Rs. Standard errors in 6a-6b based on bootstrap estimations (1000 iterations). Returns per hectare of soybean production calculated based on soybean values on or about March 1^st^ in Primavera do Leste; Production costs based on data by CONAB, also for Primavera do Leste.

In Fig 6a and 6b we plot θ for each year estimated. For change in employment, our estimates of θ vary from 0.08 (in 2006) to 0.41 (in 2004), in essence suggesting that each square kilometer of agriculture contributed to the creation of between 0.08 and 0.41 new formal sector jobs per year, depending on the year in question. Our estimates of θ for changes in non-agricultural GDP also vary by year, ranging from a high of 73,000$Rs in 2008, to negative numbers during the middle of the past decade.

In certain cases the annual variation in the estimated values for θ might suggest instability in the estimation procedure. However, here the variation in our estimates of θ closely tracks the last decade of agricultural returns. For example, in our specifications of change in employment, we estimated that θ was highest during 2002–2004, and in 2008–2009, when soybean returns were highest. Conversely, we estimated that θ was negligible during the mid-2000s, when returns were at their lowest. We bring this pattern into striking relief in Fig 6a and 6b, where we contrast our estimates of θ with the last decade of agricultural returns. Effectively, our estimations show soybean agriculture generated new employment and non-agricultural GDP increases in years when returns to production are high. During years when returns were poor, agriculture had minimal or zero impact on growth in non-agricultural employment. This is largely in line with the theoretical positions outlined in the initial sections of this article, namely that agriculture drives demand for services or consumption goods, or is capable of serving as a supplier of capital investments to the non-agricultural sector.

### Specification Set 4: Decade Changes

Finally, we test a fourth set of specifications, regressing changes in non-agricultural GDP and formal sector employment over the course of the last decade on neighborhood agriculture and the set of local control variables. For non-agricultural GDP and non-agricultural, formal sector employment, we use total change between 2001 and 2010. For nighttime light emissions, we use the change in nighttime lights between 2001 (satellite F15), and 2010 (satellite F18). For urban population change, we use changes between 2000 and 2010 the two census years. In each regression we use total neighborhood agriculture from year 2001, the first year available.

We estimate three tests for each dependent variable. First, we include, in addition to agriculture, only a lag and squared lag of the variable in question (e.g., formal employment and formal employment squared, in 2001). Second, in addition to the lagged socioeconomic variables, we include a full suite of control variables. Third, we omit the variable lags, and regress change in each socioeconomic variable on neighborhood agriculture and the set of controls, only. We present the results of each of these estimations in Table 3.

**Table 3:** OLS estimation results, specification set 4.

	Table 3											
	*Change in* EMPLOYMENT			*Change in* GDP *(1*,*000s $Rs)*			*Change in* NIGHT LIGHTS			*Change in* URB. POPULATION		
	Test 1	Test 2	Test 3	Test 1	Test 2	Test 3	Test 1	Test 2	Test 3	Test 1	Test 2	Test 3
LDV	0.777	0.717	No	0.494**	0.521**	No	-0.13	-0.14	No	0.168	0.165	No
LDV2	-0.00	0.94	No	0.00**	0.00*	No	0.00	0.00	No	0.00	0.00	No
**ΔAgriculture** ^**N**^	1.59**	2.00**	2.31**	135**	148*	211.5**	0.40**	0.53**	0.58**	5.26**	**6.8**	8.07**
Elevation^**N**^	No	-3.067	-4.18	No	8.87	-183	No	-0.515	-0.77	No	-5.23	-12.89
Slope^**N**^	No	660	488	No	-25.212	10,311	No	-36	-12	No	-1,640	-298
Km to São Paulo	No	0.22	0.02	No	44	-39	No	-0.048	-0.12	No	0.70	-0.550
Km to Major River	No	-2.49	-2.67	No	23	9.5	No	-1.22	-1.41	No	-3.48	5.8
Km to Major Road	No	-0.75	0.13	No	18**	84.83	No	0.48	0.24	No	-0.25	-1.97
Open Land^**N**^	No	-0.08	0.14	No	-124	12.68	No	0.02	-0.01	No	-0.51	0.13
Soil Type^**N**^	No	Yes	Yes	No	Yes	Yes	No	Yes	Yes	No	Yes	Yes
Soil Texture^**N**^	No	Yes	Yes	No	Yes	Yes	No	Yes	Yes	No	Yes	Yes
Cons	-33.90	858	1,515	1,505	47,413	179,172	124	516	633	-655.273	8,502	14,245
R2	0.39	0.47	0.32	0.90	0.91	0.37	0.61	0.66	0.43	0.80	0.85	0.41
**N**	**139**			**137**			**139**			**124**		
**Time Period**	**2001–2010**			**2001–2010**			**2001–2010**			**2000–2010**		

LDV = lagged dependent variable.

For employment, GDP, or night lights, figures lagged variables correspond to levels in 2001; for urban population, year 2000.

^**N**^ denotes neighborhood variable, e.g., total (for agriculture and non-forest), mean (elevation and slope), or mode (for soil variables) within a city’s sixty minute neighborhood.

**: p <. 01

*: p <. 05 Significance based on bootstrapped standard errors (1,000 iterations, seeded at 1)

The results of this fourth set of specifications indicate that agriculture has had a positive and significant impact on each of the four socioeconomic variables. In our employment regressions we estimate that each square kilometer of agriculture in 2001 contributed, over the past decade, to the creation of between 1.59 and 2.3 new, formal sector jobs outside of the agricultural sector (depending on the specification). For non-agricultural GDP, we estimate that each square kilometer of agriculture in 2001 contributed to between 135,000$Rs and 212,000$Rs in growth in GDP. We also estimate positive impacts in terms of nighttime light increases, and in urban population.

**Fig 7:**
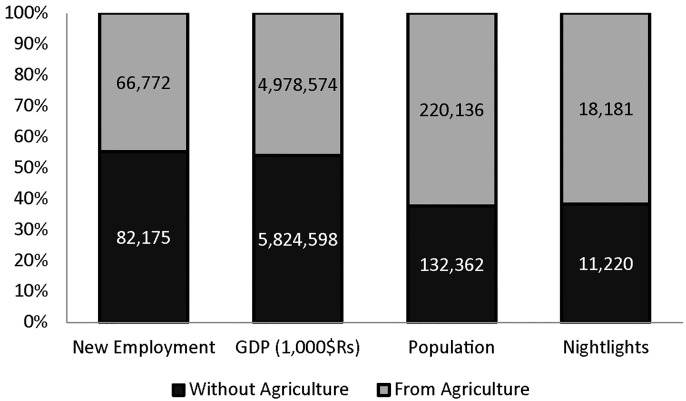
Total predicted and simulated growth. Predicted and simulated growth in each socioeconomic variable, indicating the impact of Mato Grosso’s agriculture sector.

To put our estimates into better context, we use our estimates from the second test in the fourth set of specifications (e.g., we include the lagged socioeconomic variables, neighborhood agriculture, and the full set of controls) to simulate total change in each socioeconomic variable over the past decade that is attributable to the state’s agricultural sector. We complete these simulations by first predicting change in each socioeconomic variable using the full set of control parameters from test 2, then predicting total change in a scenario where agricultural area in 2001 is zero, and by comparing the differences. We show the results in Fig 7.

In total, we estimate that the agricultural sector contributed to the creation of more than 67,000 new, formal sector jobs outside of agriculture. This amounts to approximately fifty-five percent of predicted, total non-agricultural, formal sector employment generated during the decade. We also estimate that agriculture contributed to forty-five percent of the increase in non-agricultural GDP in Mato Grosso (excluding the capital region), or nearly 5 billion $Rs. In 2010 values, this amounted to approximately 9 billion $Rs, or about 5.5 billion $US. Finally, we estimate that agriculture accounted for approximately sixty-five percent of nighttime light emissions and urban population growth in Mato Grosso between the two censuses, or more than 200,000 new residents in the region.

## Discussion and Conclusions

In this research we show that local, rural soybean production has had a clear and positive impact on regional growth in nighttime light emissions, non-agricultural GDP, urban population, and formal sector employment. Specifically, we show that approximately forty-five percent of the state’s growth in non-agricultural GDP and more than half of the formal sector, non-agricultural employment, growth in urban population and nighttime light emissions, was tied to the state’s agricultural sector. We also find that the impact of agriculture on economic and employment growth in the state was closely tied to returns to agricultural production.

Given these findings, we (a) argue that export-oriented agriculture, under certain conditions, may have an important role in generating non-agricultural employment and economic growth. However, we caution that (b) this development has already come at a heavy cost to the region’s environment, and (c) policy makers seeking to replicate the development path of Mato Grosso should recognize the institutional conditions that have facilitated agriculturally-driven growth in this region. In our discussion we expand on each of these topics.

### Export Agriculture and Economic Growth

In this research we showed that economic growth in Mato Grosso’s non-agricultural sector was closely tied to the expansion of soybean agricultural systems. These results build on observations that Mato Grosso’s soybean producing regions already rank among the highest in northern Brazil in terms of the UN’s human development indices (HDI) [34], as well as evidence that soybean production in these areas has contributed to reduced poverty, better schools, and (negatively) increased inequality [12,13].

Economic growth in Mato Grosso, however, has largely occurred without large-scale industrialization or the growth of a local manufacturing sector. Instead, growth here has mirrored the trend toward service sector-led economic growth that has been visible in other regions of the world. Most notably, research to date has suggested that in oil rich nations of Africa (Nigeria), the Middle East (Qatar, Dubai) or Latin America (e.g., Venezuela), development and urbanization has occurred as urban migrants take part in either the extraction of valuable commodities, or in servicing the labor and capital involved in the process[28]. For Mato Grosso, the past decade of economic growth has not been fueled by the extraction of minerals (although it is clearly tied to phosphorus and potash imports from North Africa, Canada, and Asia). Rather, growth here has been propelled by exports to the increasingly wealthy and urbanized middle classes of the Eastern Europe, the Middle East, and East Asia. As with any region dependent primarily on petroleum or other natural resources as a principal source of foreign exchange, or on the critical imported resource inputs needed to produce these goods, this development pathway will continue to be susceptible to swings in global prices, or in currency values.

In Mato Grosso, as we showed in Fig 6a and 6b, future economic growth will depend on returns to soybean production. Given that these returns are largely exogenous, and outside of the control of local policy makers, the local economy will recess or grow only as production returns permit. A bumper harvest in the American Midwest, or a decline in economic growth in China could depress global prices, and in turn, lead to an economic slowdown in Mato Grosso. Conversely, another precipitous decline in the real exchange rate with the US dollar might result in a boom for urban growth in Mato Grosso.

### Is the Mato Grosso case generalizable?

Mato Grosso, as an agricultural state, has benefitted from a number of geographic and institutional conditions that have increased the capacity of the state’s agriculture sector to serve as a growth engine. First, Mato Grosso is a relatively recent agricultural frontier. Even today, properties are still being cleared or converted to agriculture as investment capital is made available. Cities, and specifically those located in the midst of agriculture, are also growing rapidly, largely fueled by in-migration from elsewhere in Brazil. In time, returns to agriculture may no longer be sufficient to continue to drive local, non-agricultural growth, and future growth must come through endogenous demand, or through other economic sectors.

Second, Mato Grosso’s agricultural sector is tightly coupled with local, urban-based supply chains [20]. Were soybeans planted using inputs sourced directly from one of Brazil’s ports, and shipped from the region without being stored or processed (or fed to poultry, etc), less capital would be captured, and remain locally. However, given both the strength of the local supply chain, and the presence of downstream processing facilities in Mato Grosso, the local economy captures and circulates a larger proportion of the potential value of each harvest.

Third, and finally, in Mato Grosso, many farm owners live and spend locally [35]. Consequently, farmers will be more likely to reinvest their profits locally. Were farm owners, or the skilled workers who manage the farms and operate the machinery, to reside out of the region (for example in São Paulo or abroad) a lesser proportion of the capital generated through agriculture would be circulated locally or channeled into local non-agricultural investments. Consequently, policymakers seeking to maximize the benefits of a growing agriculture or resource sector based on export demand must ensure that the proportion of extracted value that is captured or circulated regionally is maximized.

### The cost of development

We close by recognizing that tropical agriculture has been widely identified as a driver of environmental change, and has been linked broadly to deforestation [36–38]. Not coincidentally, during the peak years of soybean profitability (2002-early 2004) deforestation in Mato Grosso reached new heights. From 2001–2006 nearly 60,000km² of tropical Amazon forest were cleared in the state; approximately ten percent of these clearings were converted directly to agriculture [3]. From 2000 to 2010, across the entirety of Brazil’s Amazon region, more than 200,000km² of forest, were razed.

There is no clear method by which to value the forest lost in the Amazon, or in Mato Grosso specifically[39]. However, using only the price of carbon as a measure of value, the costs of deforestation have been substantial. As of 2006, more than 310,000km² of forest was cleared in Mato Grosso, the equivalent of approximately 4.8 billion metric tons of carbon[40]. Presently, in the EU and California carbon markets, carbon emissions are valued at approximately 10$ per ton. If the value of forest lost to agriculture and pasture were to be measured only in terms of carbon equivalent emissions, then the cost of past deforestation in Mato Grosso would amount to approximately 48 billion $US. We contrast this figure against the annual value added by the state's agricultural sector.

In the second set of specifications we estimated an annual spillover effect of approximately 210,000$US per square kilometer of agriculture. In 2010, Mato Grosso’s farmers planted 62,000km² of soybean production. We thus estimate that that year the total spillover effect amounted to approximately 13 billion $US. The agriculture sector in Mato Grosso also contributed, directly, to approximately 6.5 billion $US in gross value added[24]. Together, these direct and indirect effects of the agriculture sector amount to nearly eighty percent of the state’s economy outside, excluding the capital region. The magnitude of these effects suggest that the annual economic benefits of agricultural expansion, when extrapolated over a significant time period and weighed against the value of carbon lost in the state, surpass the value of the lost forest.

Nevertheless, the value of a forest certainly extends far beyond the value of the carbon stored under its canopies. And despite the recent, so-called, decoupling of tropical forest loss from agricultural expansion, the specter of these environmental costs remains [2,41]. Whether future growth in the agriculture sector will continue to occur through new deforestation and land clearing, or through the intensification of land use on already cleared lands will remain to be seen. However, evidence is increasingly suggesting that Brazil is following the latter path. In recent years, much of Mato Grosso’s recent growth in agricultural production has occurred through intensification on already cleared lands [42,43]. Research has also already suggested that cattle pastures, as well as agricultural systems are trending toward intensification [32,44]. Further intensification present a pathway by which Mato Grosso’s regional economy may continue to grow, while limiting further environmental costs.

## Supporting Information

S1 Appendix Table 1, Regression results for intercalibration estimations. Figs 1a–1d, Figs S1a and S1b show nighttime light values in northern Mato Grosso. Figure S1a includes the full range of pixels, including those less than eight. Figure S1b includes only values larger than eight. Figure S1c overlays urban areas from Brazil’s 2010 census over nighttime light emissions. Figure S1d shows the processed urban lights used in this analysis. Figure 2, Adjusted and Unadjusted Nighttime Light Emissions for Mato Grosso.
(DOCX)

S1 Data Supporting data. (ZIP)

S1 Manuscript Table 1, Deflation Rates. Rates used to adjust nominal GDP Values. Table 2, Mean values of time variant variables. Table 3, Summary statistics of time invariant control variables. Table 4a, Specification Set 2, Dependent Variable: Non-Agricultural Employment, 2002–2005. Table 4b, Specification Set 2, Dependent Variable: Non-Agricultural Employment, 2006–2010. Table 5a, Specification Set 2, Dependent Variable: Non-Agricultural GDP, 2002–2005. Table 5b, Specification Set 2, Dependent Variable: Non-Agricultural GDP, 2006–2010. Table 6a, Specification Set 3, Dependent Variable: Annual Change in Non-Agricultural Employment, 2002–2005. Table 6a, Specification Set 3, Dependent Variable: Annual Change in Non-Agricultural Employment, 2006–2010. Table 7a, Specification Set 3, Dependent Variable: Annual Change in Non-Agricultural GDP, 2002–2005. Table 7a, Specification Set 3, Dependent Variable: Annual Change in Non-Agricultural GDP, 2006–2010. Table 8, Specification Set 1, IV estimations. Panel data using fixed effects, 2001–2010. Estimations using both actual and predicted agricultural areas. Table 9, Specification Set 2, IV estimations. Dependent Variable: Non-Agricultural Employment, 2001–2010. Estimates using both actual and predicted agricultural areas. Table 10, Specification Set 3, IV estimations. Dependent Variable: Change in Non-Agricultural Employment, 2001–2010. Estimates using both actual and predicted agricultural areas. Figure 1a, Estimates for θ, using a range of neighborhood sizes (in minutes, shown as x-axis. Dependent variables is formal sector, non-agricultural employment. Specifications based on test two from specification set three. Sixty minutes corresponds to the estimates of θ shown for test two in Table 3 in the principal manuscript. Figure 1b, Estimates for θ, using a range of neighborhood sizes (in minutes, shown as x-axis. Dependent variables is non-agricultural GDP. Specifications based on test two from specification set three. Sixty minutes corresponds to the estimates of θ shown for test two in Table 3 in the principal manuscript. Figure 2a, Estimates for total employment generated from agriculture, using a range of neighborhood sizes (in minutes, shown as x-axis). Predictions based on parameters from test two and specification set three. Figure 2b, Estimates for new GDP generated from agriculture, using a range of neighborhood sizes (in minutes, shown as x-axis). Dependent variables is non-agricultural GDP. Specifications based on test two from specification set three.
(DOCX)
