# Supplementary material for: Soybean Development: The Impact of a Decade of Agricultural Change on Urban and Economic Growth in Mato Grosso, Brazil
Source: PLoS One. 2015 Apr 28;10(4):e0122510. doi: 10.1371/journal.pone.0122510 (PMC4412665; doi:10.1371/journal.pone.0122510)
Supplement: S1 Appendix — Table 1, Regression results for intercalibration estimations. Figs 1a–1d, Figs S1a and S1b show nighttime light values in northern Mato Grosso. Figure S1a includes the full range of pixels, including those less than eight. Figure S1b includes only values larger than eight. Figure S1c overlays urban areas from Brazil’s 2010 census over nighttime light emissions. Figure S1d shows the processed urban lights used in this analysis. Figure 2, Adjusted and Unadjusted Nighttime Light Emissions for Mato Grosso. (DOCX) [file pone.0122510.s001.docx]

**Supplementary Information: Appendix**

**Nighttime Lights Data**

The raw satellite DMSP data is clouded by lunar reflectance and identifies deforestation fires as stable light sources. The data for each year is also not directly comparable. To correct these issues we process the data to remove lunar reflectance, cross check the light locations with known urban areas, and adjust for data for use in inter-annual and inter-satellite comparisons. Raw (e.g., average visible lights) satellite data can be downloaded at <http://ngdc.noaa.gov/eog/dmsp/downloadV4composites.html>

To correct the nighttime light data, first, we selected only pixels with values larger than eight (the full pixel range is from 0-63). We chose eight based on our understanding of where urban areas were, and the fact that observed nighttime lights in forest areas could be as high as eight. This step effectively removed lunar reflectance and other noise from our dataset (see Figures 1a-b in Appendix).


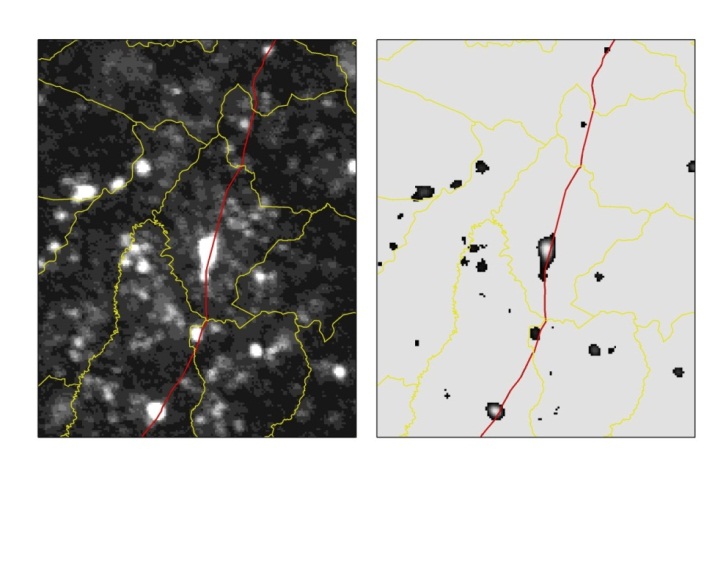

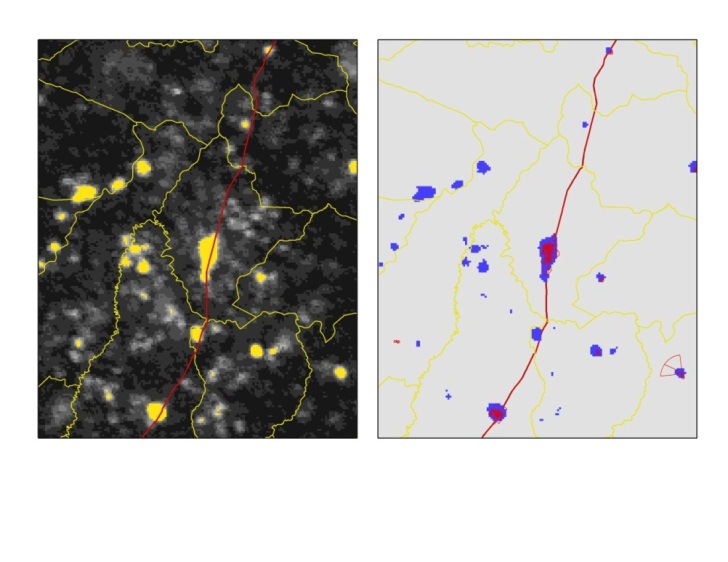

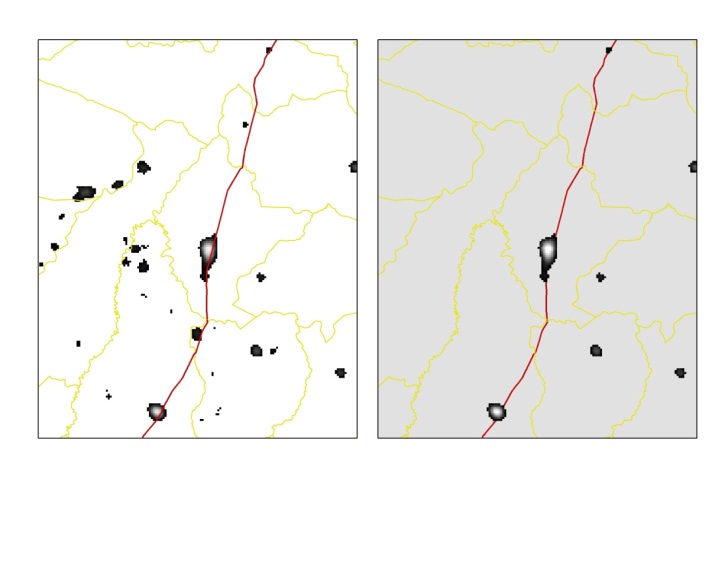


Figures 1a-1d

*Figures 1a and 1b show nighttime light values in northern Mato Grosso. Figure 1a includes the full range of pixels, including those less than eight. Figure 1b includes only values larger than eight. Figure 1c overlays urban areas from Brazil’s 2010 census over nighttime light emissions. Figure S1d shows final set of urban lights.*

To omit areas identified as nightlights, but which were likely from deforestation, we clustered all pixels with values larger than eight, and excluded any clusters not overlapping urban areas from further analysis. In Figure 1c we overlay urban areas, as classified in the 2010 census, over nighttime lights. Figures 1a and1SA2b show nighttime lights clusters before and after removing nighttime lights due to deforestation.

The final step was to inter-calibrate the nighttime lights data across satellites and data years. Following Elvidge, et al. (2009), we inter-calibrated the data against satellite F12 for year 1999. We accomplished this by regressing data values and values squared (see Equation S1) for each point from F12 in 1999 in the combined areas of Alta Floresta and Barra dos Garças (the two slowest growing cities in the state) against values for these areas from other years. We then store the coefficients, which we use to adjust the full dataset. Again, following Elvidge, et al (1999) we thus adjust the nighttime lights data as follows:

$$N_{adjusted}= B_{0}+ B_{1}N+B_{2}N^{2}$$

(equation A1)

where N refers to the nightlight value, *B_0_*, *B_1_*, and *B_2_* are estimated coefficients. We report the coefficients for each regression in Table 1 in the Appendix. In Figure3 we show the results of the adjustment process as the evolution of total nighttime lights in Mato Grosso, both before and after adjustment. The point values grow steadily in the adjusted values, and inter-satellite variation is diminished.

| **Table 1 in Appendix** | | | | |  |
| --- | --- | --- | --- | --- | --- |
| Regression results for inter-calibration estimations. | | | | |  |
|  |  |  |  |  |  |
| Sat | Year | Β_0_ | Β_1_ | Β_2_ | R2 |
| F18 | **2010** | 0.010416 | **0.65407** | 0.001243 | 0.7782 |
| F16 | **2009** | 0.020548 | **1.00224** | -0.00182 | 0.8237 |
| F16 | **2008** | 0.008203 | **0.82473** | 0.003364 | 0.9325 |
| F16 | **2007** | 0.00151 | **0.81537** | 0.005734 | 0.9586 |
| F16 | **2006** | 0.006921 | 0.827019 | 0.004015 | 0.9296 |
| F16 | **2005** | 0.017733 | **1.39221** | -0.00483 | 0.908 |
| F16 | **2004** | 0.007629 | **1.18728** | -0.00103 | 0.9582 |
| F15 | **2007** | 0.01924 | **1.18816** | -0.00057 | 0.9088 |
| F15 | **2006** | 0.023573 | **1.37017** | -0.00364 | 0.8947 |
| F15 | **2005** | 0.023163 | **1.55974** | -0.00512 | 0.9249 |
| F15 | **2004** | 0.028842 | **1.66085** | -0.00896 | 0.8979 |
| F15 | **2003** | 0.035722 | **1.87673** | -0.01336 | 0.9055 |
| F15 | **2002** | 0.019159 | **1.31347** | -0.00464 | 0.8345 |
| F15 | **2001** | 0.010653 | **1.35347** | -0.00465 | 0.9667 |
| F15 | **2000** | 0.00664 | **1.08092** | -0.00103 | 0.9792 |
| F14 | **2003** | 0.019362 | **1.29697** | -0.00549 | 0.9204 |
| F14 | **2002** | 0.024209 | **1.51909** | -0.00921 | 0.9058 |
| F14 | **2001** | 0.025123 | **1.58259** | -0.00924 | 0.9194 |
| F14 | **2000** | 0.020063 | **1.29001** | -0.00442 | 0.9204 |
| F14 | **1999** | 0.018981 | **1.36197** | -0.00601 | 0.9429 |
| F14 | **1998** | 0.020102 | **1.42719** | -0.00653 | 0.9532 |
| F14 | **1997** | 0.020869 | **1.64322** | -0.00982 | 0.921 |
| F12 | **1999** |  | **1** |  | 1 |
| F12 | **1998** | 0.004265 | **0.94786** | 0.000811 | 0.9629 |
| F12 | **1997** | 0.012014 | **1.29611** | -0.00399 | 0.9313 |
| F12 | **1996** | 0.017259 | **1.43895** | -0.00662 | 0.9249 |
| F12 | **1995** | 0.011595 | 1.217203 | -0.00187 | 0.8872 |
| F12 | **1994** | 0.015651 | **1.26437** | -0.00382 | 0.8031 |
| F10 | **1994** | 0.035538 | 1.943407 | -0.01717 | 0.8861 |
| F10 | **1993** | 0.037226 | **2.14543** | -0.02067 | 0.8976 |
| F10 | **1992** | 0.054726 | **1.61937** | -0.01449 | 0.6952 |

Figure 2

Total nighttime light emissions both before and after data adjustment.
