# Supplementary material for: Soybean Development: The Impact of a Decade of Agricultural Change on Urban and Economic Growth in Mato Grosso, Brazil
Source: PLoS One. 2015 Apr 28;10(4):e0122510. doi: 10.1371/journal.pone.0122510 (PMC4412665; doi:10.1371/journal.pone.0122510)
Supplement: S1 Manuscript — Table 1, Deflation Rates. Rates used to adjust nominal GDP Values. Table 2, Mean values of time variant variables. Table 3, Summary statistics of time invariant control variables. Table 4a, Specification Set 2, Dependent Variable: Non-Agricultural Employment, 2002–2005. Table 4b, Specification Set 2, Dependent Variable: Non-Agricultural Employment, 2006–2010. Table 5a, Specification Set 2, Dependent Variable: Non-Agricultural GDP, 2002–2005. Table 5b, Specification Set 2, Dependent Variable: Non-Agricultural GDP, 2006–2010. Table 6a, Specification Set 3, Dependent Variable: Annual Change in Non-Agricultural Employment, 2002–2005. Table 6a, Specification Set 3, Dependent Variable: Annual Change in Non-Agricultural Employment, 2006–2010. Table 7a, Specification Set 3, Dependent Variable: Annual Change in Non-Agricultural GDP, 2002–2005. Table 7a, Specification Set 3, Dependent Variable: Annual Change in Non-Agricultural GDP, 2006–2010. Table 8, Specification Set 1, IV estimations. Panel data using fixed effects, 2001–2010. Estimations using both actual and predicted agricultural areas. Table 9, Specification Set 2, IV estimations. Dependent Variable: Non-Agricultural Employment, 2001–2010. Estimates using both actual and predicted agricultural areas. Table 10, Specification Set 3, IV estimations. Dependent Variable: Change in Non-Agricultural Employment, 2001–2010. Estimates using both actual and predicted agricultural areas. Figure 1a, Estimates for θ, using a range of neighborhood sizes (in minutes, shown as x-axis. Dependent variables is formal sector, non-agricultural employment. Specifications based on test two from specification set three. Sixty minutes corresponds to the estimates of θ shown for test two in Table 3 in the principal manuscript. Figure 1b, Estimates for θ, using a range of neighborhood sizes (in minutes, shown as x-axis. Dependent variables is non-agricultural GDP. Specifications based on test two from specification set three. Sixty minutes corre [file pone.0122510.s003.docx]

**Supplemental Materials**

**Data**

**Employment**

For our analysis we use total employment in four sectors: (a) services and restaurants, (b) public employees, health, and education, (c) construction, and (d) commerce and sales. We focus on these four sectors, as we identified employees in these sectors as more likely to be based in urban areas. Employment data was downloaded from the Relação Anual de Informações Sociais (RAIS, Annual Relation of Social Information) website [www.rais.br].

**Non-agricultural GDP**

For GDP, we downloaded county (municipio) data on total GDP total GDP added through agriculture and ranching from the from the Brazilian Institute of Geography and Statistics [IBGE] data clearinghouse, Sidra [www.sidra.ibge.gov.br]. We calculated non-agricultural GDP as total GDP minus GDP from agriculture and ranching. We then adjusted GDP to 2001 currency values by calculating an adjustment rate based on World Bank Deflator rates [see Table S1]. Adjusted values equal non-agricultural GDP, divided by the adjustment rate below.

| Table S1  Deflation Rates | | |
| --- | --- | --- |
| Year | World Bank Deflator | Calculated Adjustment Rate |
| 2001 | 1 | 1 |
| 2002 | 10.6 | 1.106 |
| 2003 | 13.7 | 1.243 |
| 2004 | 8 | 1.323 |
| 2005 | 7.2 | 1.395 |
| 2006 | 6.2 | 1.457 |
| 2007 | 5.9 | 1.516 |
| 2008 | 8.3 | 1.599 |
| 2009 | 7.2 | 1.671 |
| 2010 | 8.2 | 1.753 |
| 2011 | 7 | 1.823 |

**Population**

We use urban population data from Brazil’s 2000 and 2010 censuses. Population data is available only during the census years, thus we only test population change between the two census periods.

**Nighttime Lights**

For nighttime lights we used the 30 arc second resolution (approximately 1km²) Defense Meteorological Satellite Program (DMSP) data on global nighttime light emissions [27]. In the DMSP data each pixel is valued at between zero and sixty three. Sixty-three signifies maximum light output, while zero is no lights. We use the raw, unprocessed DMSP dataset, then execute several steps of processing (see appendix in SI) to correct for lunar reflectance and fires from deforestation, and to calibrate the data for cross year and cross satellite comparisons (e.g., following Elvidge, et al 2009 [45])

The advantage of the DMSP nightlight data, as a measure of human population, lay in its its spatial resolution. The finer resolution of the dataset also enables us to pinpoint the location of urban populations, and to examine population without relying on politically drawn census tracts. We also note that nighttime light emissions are already widely used as a measure of urban growth in quantitative and spatially explicit studies of urbanization [23-26]. Nighttime light emissions are also highly correlated with urban population data. For example, we estimate a 96 percent correlation between nighttime light emissions in 2000 and 2010 and urban population counts in the 2000 and 2010 censuses.

**Agricultural Data**

Agricultural area is based on land use classifications using MODIS satellite images and crop phonologies [32,39]. The land use classifications identify single and double cropping, as well as crop type (e.g., soybeans, soybean-corn, soybean-cotton, soybean-other, or cotton). For our analysis we focus only on agricultural area. Each pixel is 1km². As described below, in the section entitled neighborhoods, the key variable used in this analysis is the sum of agriculture area in each city’s neighborhood. Mean values for each of the dependent variables and agricultural area are included in table S2.

**Control Variables**

Aside from agricultural area, we use eight additional control variables. Four distance variables were calculated in a GIS (km to São Paulo, nearest federal highway (built before 1985), and major river). We acquired elevation from the NASA’s Shuttle Radar Topography Mission (SRTM), which we then used to calculate slope. Finally, we estimate non-forest area from Hansen, et al’s 2013 Global Forest Maps [33]. We classify any areas with less than fifty percent forest cover as non-forest, then calculated forest cover for each year as total area not in forest in 2000, plus total area deforested for each additional year. Finally, we use soils data acquired from Embrapa, the Brazilian Agricultural Research Corporation, a research institute associated with the Brazilian Government. As with the agricultural data, we calculate total non-forest area as the total within each city’s neighborhood. We also calculated the most common soil type and texture, and the mean slope and elevation. Descriptive statistics for all control variables are included in Tables S2 and S3.

|  | | Table S2  Mean values of time variant variables. | | | | | | | |
| --- | --- | --- | --- | --- | --- | --- | --- | --- | --- |
| Year | Employment | | Non-Ag GDP^+^ | Nightlights (F15) | Nightlights (F16) | Nightlights (F18) | Urban Population | Agricultural Area (km²) | Open Land (km²) |
| 2000 | · | | · | · | · | · | 10,482 | · | · |
| 2001 | 855.6 | | 55,023 | 496.8 | · | · | · | 247.7 | 2,491 |
| 2002 | 949 | | 70,661 | 536.4 | · | · | · | 253.3 | 2,537 |
| 2003 | 1,060 | | 93,850 | 531.5 | · | · | · | 277.8 | 2,581 |
| 2004 | 1,155 | | 116,259 | 550.2 | 663.6 | · | · | 313.3 | 2,631 |
| 2005 | 1,210 | | 120,884 | 586.2 | 563.6 | · | · | 356.4 | 2,658 |
| 2006 | 1,263 | | 125,213 | 559.4 | 589.2 | · | · | 352.7 | 2,677 |
| 2007 | 1,375 | | 150,369 | 562.2 | 614.1 | · | · | 305 | 2,694 |
| 2008 | 1,513 | | 190,862 | · | 611.4 | · | · | 381.6 | 2,709 |
| 2009 | 1,634 | | 205,506 | · | 618.6 | · | · | 371.8 | 2,718 |
| 2010 | 1,750 | | 232,422 | · | · | 728.3 | 12,181 | 343.9 | 2,735 |
|  | | ^+^ GDP values in 1,000$Rs | | | | | | | |

| Table S3 |  |  |  |  |  |
| --- | --- | --- | --- | --- | --- |
| Summary statistics of time invariant control variables | | | | | |
|  |  | (2) | (3) | (4) | (5) |
|  |  | mean | sd | min | max |
|  |  |  |  |  |  |
| Mean Elevation^N^ | | 362.1 | 97.54 | 147.8 | 651.7 |
| Mean Slope^N^ | | 0.848 | 0.349 | 0.0914 | 1.623 |
| Km to São Paulo | | 1,442 | 261.8 | 905.2 | 2,136 |
| Km to Major River | | 25.64 | 22.60 | 0 | 110.1 |
| Km to Major Road | | 70.38 | 70.04 | 0 | 310.1 |
|  |  |  |  |  |  |

**Neighborhoods.** A key component of this research is the definition of each city’s neighborhood. To accomplish this we identify neighborhood areas as a function of travel times between each city and its rural surroundings. We begin this process by overlaying a fishnet of 5km² grid cells (*n=~30,*000) across the extent of Mato Grosso State. We then discarded protected areas and those that were more than twenty kilometers away from any roadway, leaving a total of ~26,000 grid cells.

Next, we estimated total travel times between each city and its surrounding areas, using the network analyst toolset in ArcGIS to measure travel in time between each grid center point and each city center. To calculate travel times, we first estimated travel times for each road segment. To accomplish this, we first assigned a road speed for each segment based on its attributes. Two lane highways were classified as 150km/h, paved roads as 100km/h, unpaved roads at 50km/h, unpaved seasonal roads at 25km/h, bridges at 10km/h, and ferry crossing as 1km/h. These speeds approximate the lead author’s own driving times on the corresponding road types. Next, we calculated a travel time for each segment as a function of its length and the time needed to complete it, given the corresponding speed. ArcGIS’s network analyst then estimated the shorted path between each city and each grid centroids, based on the network of road segments and travel times.

We first set a neighborhood threshold of sixty minutes. However, we recognize that this threshold is arbitrary. Consequently, as a sensitivity test, we compare our estimated parameters from a sixty minute threshold with those estimated under a range of neighborhood sizes, from twenty to three hundred minutes (the results are included in the SI, under sensitivity tests).

To calculate neighborhood agriculture we summed the total area classified as agriculture within each 5km grid cell. Total areas ranged from zero to 25 (e.g., 25 1km² pixels). Total neighborhood agriculture then refers to the sum of agriculture from all pixels classified as pertaining to each city’s neighborhood. For the soil variables, we classified each 5km grid cell as whatever soil type was located under its center point, and then identified the mode within each grid cell from the range of grid cells in each neighborhood. For slope and elevation we estimated the mean of each variable for each 5km grid cell, then used these measures to estimate the mean across the set of 5km grid cells each city’s entire neighborhood. Similarly, for non-forest area, we first estimated the total area in each 5km grid cell, then calculated the sum of non-forest area from each grid cell pertaining to a city’s neighborhood.

**Specifications**

**Specification Set 1**

In specification set one we regress each urban socioeconomic variable, *U* for each year, *t* and city, *i*, as a function of one year lagged area of agriculture (*A*) in a fixed effects model. In this specification (equation S1) we include a group effect, shown as *c*, to control for spatially heterogeneous, time-static attributes for each city, and year effects to control for year-varying effects (shown as *y*). We also include total area of non-forest, which changes by year (*O*). We write the resulting specification as:

$(U_{i,t})= c_{i}+y_{t}+ \theta A_{i,t-1}+\beta_{3}O_{i,t-1}+ \varepsilon_{i,t}$ (S1)

The results of specification set 1 are included in the main article.

**Specification Set 2**

The objective of the second set of specifications is to estimate *U* as a function of urban areas’ surrounding agricultural production, for each year of our panel set. Thus rather than estimate a single coefficient for θ over the full extent of our data (as in Specification Set 1) we specified our estimates for each year. The objective of this specification set was to test for stability in our estimates over time, or observes patterns in our estimates. We accomplish this objective by estimating *U* for each year and city as:

$(U_{i,t})= \beta_{0}+ \theta A_{i,t-1}+\beta_{3}X_{i,t-1}+ \varepsilon_{i,t}$ (S2)

Where each urban socioeconomic variable (shown as *U*) is regressed on neighborhood agriculture and a vector of eight control variables (shown as *X*), including slope, elevation, non-forest area, and locational variables. Again, the variable of key interest to our analysis is agricultural area in each city’s neighborhood. We show this as *A* in equation S2. In our results, we focus on our estimates of *θ*, which we expect to be positive and significant. Owing to data limitations, and the difficulty of comparing the nighttime light data across multiple time periods, we do not specify urban population or nighttime light emissions in Specification Sets 2 or 3. The full results are included as Tables S4-S5.

| **Table S4a**  **Specification Set 2.**  **Dependent Variable: Non-Agricultural Employment, 2002-2005** | | | | | |
| --- | --- | --- | --- | --- | --- |
|  | **2005** | **2004** | | **2003** | **2002** |
| **Agriculture_t-1_^N^** | **1.741** | | **1.700** | **1.218** | **0.734** |
|  | **(0.901)** | | **(0.795)*** | **(0.913)** | **(0.573)** |
| Mean Elevation^N^ | -5.545 | | -5.140 | -3.968 | -2.381 |
|  | (3.771) | | (3.752) | (3.449) | (2.569) |
| Mean Slope^N^ | 953.933 | | 894.319 | 537.662 | 260.909 |
|  | (829.394) | | (716.997) | (751.090) | (710.730) |
| Km São Paulo | -0.577 | | -0.424 | -0.476 | -0.488 |
|  | (1.166) | | (1.018) | (0.995) | (0.790) |
| Km to Main River | -2.643 | | -2.680 | -1.824 | -1.550 |
|  | (7.069) | | (6.769) | (7.044) | (5.392) |
| Open Area**_t-1_^N^** | 0.269 | | 0.280 | 0.297 | 0.307 |
|  | (0.129)* | | (0.120)* | (0.108)** | (0.113)** |
| Km to Main Road | -0.411 | | -1.020 | -0.571 | 0.124 |
|  | (3.528) | | (3.296) | (2.655) | (2.400) |
| Soil Type^N^ | Yes | | Yes | Yes | Yes |
| Soil Texture^N^ | Yes | | Yes | Yes | Yes |
| Constant | 2,661.436 | | 2,256.706 | 2,006.639 | 1,681.624 |
|  | (1,889.486) | | (1,739.266) | (1,654.777) | (1,393.781) |
| *R*^2^ | 0.22 | | 0.21 | 0.18 | 0.17 |
| *N* | 139 | | 139 | 139 | 139 |

* *p*<0.05; ** *p*<0.01

| **Table S4b**  **Specification Set 2.**  **Dependent Variable: Non-Agricultural Employment, 2006-2010** | | | | | | | | |  |
| --- | --- | --- | --- | --- | --- | --- | --- | --- | --- |
|  | **2010** | **2009** | | **2008** | **2007** | | | **2006** |  |
| **Agriculture_t-1_^N^** | **2.514** | | **2.300** | **2.140** | | **1.724** | **1.553** | | |
|  | **(0.517)**** | | **(0.499)**** | **(0.471)**** | | **(0.637)**** | **(0.561)**** | | |
| Mean Elevation^N^ | -7.289 | | -7.406 | -5.994 | | -5.321 | -5.597 | | |
|  | (3.716) | | (3.518)* | (3.344) | | (3.893) | (3.167) | | |
| Mean Slope^N^ | 1,213.618 | | 1,271.746 | 1,051.867 | | 903.080 | 1,028.481 | | |
|  | (1,065) | | (942.407) | (1,094.021) | | (847.493) | (822.051) | | |
| Km São Paulo | -0.4598 | | -0.330 | -0.354 | | -0.425 | -0.529 | | |
|  | (1.53) | | (1.381) | (1.205) | | (1.182) | (1.152) | | |
| Km to Main River | -7.0923 | | -6.691 | -6.372 | | -4.693 | -2.765 | | |
|  | (10.694) | | (9.808) | (8.559) | | (8.699) | (8.126) | | |
| Open Area**_t-1_^N^** | 0.416 | | 0.363 | 0.405 | | 0.356 | 0.313 | | |
|  | (0.178) | | (0.162)* | (0.150)** | | (0.143)* | (0.129)* | | |
| Km to Main Road | 0.960 | | 0.427 | 0.524 | | 0.285 | -0.440 | | |
|  | (3.515) | | (3.132) | (3.712) | | (2.638) | (2.737) | | |
| Soil Type^N^ | Yes | | Yes | Yes | | Yes | Yes | | |
| Soil Texture^N^ | Yes | | Yes | Yes | | Yes | Yes | | |
| Constant | 3,301.955 | | 3,071.899 | 2,804.340 | | 2,689.718 | 2,706.162 | | |
|  | (2,533.403) | | (2,304.874) | (2,028.928) | | (1,964.139) | (1,964.283) | | |
| *R*^2^ | 0.28 | | 0.27 | 0.25 | | 0.24 | 0.23 | | |
| *N* | 139 | | 139 | 139 | | 139 | 139 | | |

* *p*<0.05; ** *p*<0.01

| **Table S5a**  **Specification Set 2.**  **Dependent Variable: Non-Agricultural GDP, 2002-2005** | | | | | |
| --- | --- | --- | --- | --- | --- |
|  | **2005** | **2004** | | **2003** | **2002** |
| **Agriculture_t-1_^N^** | **125.538** | | **141.636** | **111.824** | **80.380** |
|  | **(91.513)** | | **(64.196)*** | **(39.312)**** | **(47.551)** |
| Elevation^N^ | -204.535 | | -161.233 | -125.928 | -135.256 |
|  | (367.978) | | (216.066) | (175.550) | (143.017) |
| Mean Slope^N^ | 43,590.018 | | 30,392.239 | 22,049.680 | 24,910.196 |
|  | (61,764.700) | | (41,836.825) | (41,387.639) | (32,303.736) |
| Km São Paulo | -33.781 | | -17.148 | -17.856 | -5.639 |
|  | (74.627) | | (74.331) | (56.568) | (49.976) |
| Km to River | -14.747 | | -158.508 | -161.519 | -89.630 |
|  | (469.970) | | (431.238) | (344.440) | (391.705) |
| Open Area**_t-1_^N^** | 22.371 | | 22.031 | 20.253 | 19.995 |
|  | (8.635)** | | (8.912)* | (7.664)** | (6.503)** |
| Km to Road | 88.995 | | 74.319 | 58.961 | 26.972 |
|  | (217.463) | | (186.361) | (178.168) | (121.469) |
| Soil Type^N^ | Yes | | Yes | Yes | Yes |
| Soil Texture^N^ | Yes | | Yes | Yes | Yes |
| Constant | 118,153.111 | | 136,045.233 | 122,471.238 | 102,483.647 |
|  | (132,637.750) | | (133,351.117) | (112,416.526) | (144,551.770) |
| *R*^2^ | 0.26 | | 0.26 | 0.25 | 0.26 |
| *N* | 139 | | 137 | 137 | 137 |

* *p*<0.05; ** *p*<0.0

| **Table S5b**  **Specification Set 2.**  **Dependent Variable: Non-Agricultural GDP, 2010-2006** | | | | | | | | |  |
| --- | --- | --- | --- | --- | --- | --- | --- | --- | --- |
|  | **2010** | **2009** | | **2008** | **2007** | | | **2006** |  |
| **Agriculture_t-1_^N^** | **205.181** | | **205.475** | **216.482** | | **146.425** | **99.610** | | |
|  | **(38.202)**** | | **(45.148)**** | **(33.374)**** | | **(58.447)*** | **(51.237)** | | |
| Elevation^N^ | -330.572 | | -423.269 | -354.140 | | -247.145 | -158.858 | | |
|  | (339.409) | | (329.430) | (301.968) | | (329.948) | (254.682) | | |
| Mean Slope^N^ | 50,019.409 | | 68,995.751 | 60,349.349 | | 38,925.248 | 26,656.814 | | |
|  | (70,116.733) | | (54,213.932) | (70,297.987) | | (55,943.307) | (46,598.278) | | |
| Km São Paulo | -60.318 | | -43.484 | -46.442 | | -43.021 | -34.576 | | |
|  | (96.529) | | (92.055) | (85.111) | | (69.861) | (57.275) | | |
| Km to River | -339.847 | | -103.219 | -147.113 | | -104.877 | -76.642 | | |
|  | (692.354) | | (689.775) | (622.572) | | (578.929) | (461.120) | | |
| Open Area**_t-1_^N^** | 37.216 | | 31.796 | 32.521 | | 25.698 | 24.957 | | |
|  | (11.579)** | | (11.795)** | (10.883)** | | (9.586)** | (7.617)** | | |
| Km to Road | 208.849 | | 149.269 | 120.865 | | 113.171 | 94.092 | | |
|  | (291.458) | | (260.187) | (250.227) | | (207.284) | (157.923) | | |
| Soil Type^N^ | Yes | | Yes | Yes | | Yes | Yes | | |
| Soil Texture^N^ | Yes | | Yes | Yes | | Yes | Yes | | |
| Constant | 197,534.088 | | 193,129.490 | 172,183.394 | | 146,976.464 | 110,813.847 | | |
|  | (146,312.669) | | (148,607.857) | (129,182.771) | | (103,301.979) | (82,409.025) | | |
| *R*^2^ | 0.32 | | 0.31 | 0.31 | | 0.28 | 0.26 | | |
| *N* | 139 | | 139 | 139 | | 139 | 139 | | |

* *p*<0.05; ** *p*<0.01

**Specification Set 3**

The objective of the third set of specifications is to estimate urban socioeconomic *change* as a function of urban areas’ surrounding agricultural production, in one year intervals. We accomplish this objective by estimating *change* in *U* as:

$(U_{i,t}-U_{i,t-1})= \beta_{0}+ \theta A_{i,t-1}+\beta_{3}X_{i,t-1}+ \varepsilon_{i,t}$ (S3)

Where change in *U* for each time interval between *t* and *t-1* (in this case *t* equals year intervals between 2001 and 2010) for each city *i*. We also include the vector of eight control variables (shown as *X*). In our results, we focus on our estimates of *θ*, which we expect to be positive and significant, but which we also expect to vary by year, in agreement with agricultural returns. In essence, we expect that θ will be highest in years of high returns, and low in years of low or negative returns to soybean production. Full Results are included in Tables S6-S7.

| **Table S6a**  **Specification Set 3.**  **Dependent Variable: Annual Change in Non-Agricultural Employment, 2002-2005** | | | | | |
| --- | --- | --- | --- | --- | --- |
|  | **2005** | **2004** | | **2003** | **2002** |
| **Agriculture_t-1_^N^** | **0.040** | | **0.418** | **0.365** | **0.189** |
|  | **(0.113)** | | **(0.168)*** | **(0.179)*** | **(0.103)** |
| Elevation^N^ | 0.012 | | -0.705 | -1.169 | -0.282 |
|  | (0.527) | | (0.683) | (0.711) | (0.461) |
| Mean Slope^N^ | -29.229 | | 242.686 | 212.612 | 117.761 |
|  | (143.161) | | (340.483) | (182.344) | (188.027) |
| Km São Paulo | -0.077 | | 0.072 | -0.008 | -0.152 |
|  | (0.110) | | (0.242) | (0.219) | (0.154) |
| Km to River | 0.030 | | -1.049 | -0.124 | 0.107 |
|  | (0.601) | | (1.484) | (1.037) | (0.795) |
| Open Area**_t-1_^N^** | -0.006 | | -0.011 | 0.001 | 0.008 |
|  | (0.020) | | (0.060) | (0.029) | (0.030) |
| Km to Road | 0.134 | | -0.647 | -0.702 | 0.239 |
|  | (0.310) | | (0.870) | (0.539) | (0.421) |
| Soil Type^N^ | Yes | | Yes | Yes | Yes |
| Soil Texture^N^ | Yes | | Yes | Yes | Yes |
| Constant | 234.356 | | 153.107 | 271.681 | 382.319 |
|  | (196.939) | | (332.728) | (368.268) | (251.491) |
| *R*^2^ | 0.04 | | 0.19 | 0.20 | 0.16 |
| *N* | 139 | | 139 | 139 | 139 |

* *p*<0.05; ** *p*<0.01

| **Table S6b**  **Specification Set 3.**  **Dependent Variable: Annual Change in Non-Agricultural Employment, 2006-2010** | | | | | | | | |  |
| --- | --- | --- | --- | --- | --- | --- | --- | --- | --- |
|  | **2010** | **2009** | | **2008** | **2007** | | | **2006** |  |
| **Agriculture_t-1_^N^** | **0.211** | | **0.345** | **0.383** | | **0.156** | **0.083** | | |
|  | **(0.073)**** | | **(0.037)**** | **(0.059)**** | | **(0.060)**** | **(0.090)** | | |
| Elevation^N^ | -0.143 | | -0.927 | -0.864 | | -0.024 | -0.546 | | |
|  | (0.366) | | (0.280)** | (0.210)** | | (0.250) | (0.373) | | |
| Mean Slope^N^ | -37.103 | | 141.600 | 128.297 | | -17.619 | 142.034 | | |
|  | (138.618) | | (99.457) | (81.209) | | (116.090) | (136.605) | | |
| Km São Paulo | -0.023 | | -0.018 | 0.049 | | 0.133 | 0.093 | | |
|  | (0.151) | | (0.107) | (0.118) | | (0.140) | (0.150) | | |
| Km to River | -0.340 | | -0.133 | -1.204 | | -1.817 | 0.350 | | |
|  | (1.197) | | (0.835) | (0.744) | | (0.555)** | (0.766) | | |
| Open Area**_t-1_^N^** | 0.040 | | -0.008 | 0.007 | | 0.046 | 0.008 | | |
|  | (0.017)* | | (0.015) | (0.014) | | (0.019)* | (0.018) | | |
| Km to Road | 0.427 | | -0.174 | 0.244 | | 0.544 | -0.055 | | |
|  | (0.372) | | (0.314) | (0.301) | | (0.293) | (0.491) | | |
| Soil Type^N^ | Yes | | Yes | Yes | | Yes | Yes | | |
| Soil Texture^N^ | Yes | | Yes | Yes | | Yes | Yes | | |
| Constant | 162.385 | | 217.751 | 215.960 | | -51.187 | 106.089 | | |
|  | (260.528) | | (171.233) | (195.452) | | (244.903) | (309.448) | | |
| *R*^2^ | 0.31 | | 0.48 | 0.35 | | 0.38 | 0.22 | | |
| *N* | 138 | | 139 | 139 | | 139 | 139 | | |

* *p*<0.05; ** *p*<0.01

| **Table S7a**  **Specification Set 3.**  **Dependent Variable: Annual Change in Non-Agricultural GDP, 2002-2005** | | | | | |
| --- | --- | --- | --- | --- | --- |
|  | **2005** | **2004** | | **2003** | **2002** |
| **Agriculture_t-1_^N^** | -6.680 | | 27.149 | 20.474 | 21.923 |
|  | (11.589) | | (11.244)* | (11.861) | (6.432)** |
| Elevation^N^ | -26.311 | | -6.325 | 46.957 | -4.325 |
|  | (38.001) | | (39.360) | (88.160) | (35.002) |
| Mean Slope^N^ | 8,030.720 | | 521.477 | -8,502.891 | -2,993.291 |
|  | (5,894.772) | | (6,400.762) | (15,352.651) | (8,524.149) |
| Km São Paulo | -5.627 | | 1.246 | -15.047 | -9.435 |
|  | (7.798) | | (10.269) | (23.641) | (8.921) |
| Km to River | 149.414 | | -8.899 | -53.276 | 64.033 |
|  | (73.205)* | | (85.632) | (91.778) | (50.722) |
| Open Area**_t-1_^N^** | -0.484 | | 2.378 | 1.551 | 3.496 |
|  | (1.141) | | (1.596) | (2.463) | (1.285)** |
| Km to Road | -21.565 | | 2.423 | 34.553 | 27.519 |
|  | (38.163) | | (33.301) | (90.601) | (30.876) |
| Soil Type^N^ | Yes | | Yes | Yes | Yes |
| Soil Texture^N^ | Yes | | Yes | Yes | Yes |
| Constant | 15,600.609 | | 10,556.787 | 10,668.900 | 13,288.922 |
|  | (18,127.890) | | (20,683.576) | (37,686.267) | (21,594.951) |
| *R*^2^ | 0.13 | | 0.29 | 0.19 | 0.29 |
| *N* | 137 | | 137 | 137 | 137 |

* *p*<0.05; ** *p*<0.01

| **Table S7b**  **Specification Set 3.**  **Dependent Variable: Annual Change in Non-Agricultural GDP, 2010-2006** | | | | | | | | |  |
| --- | --- | --- | --- | --- | --- | --- | --- | --- | --- |
|  | **2010** | **2009** | | **2008** | **2007** | | | **2006** |  |
| **Agriculture_t-1_^N^** | 3.040 | | 9.995 | 73.455 | | 48.654 | -5.326 | | |
|  | (7.285) | | (8.595) | (10.184)** | | (13.494)** | (6.663) | | |
| Elevation^N^ | 59.985 | | -28.199 | -141.377 | | -116.308 | 6.475 | | |
|  | (67.404) | | (48.325) | (48.502)** | | (52.887)* | (36.257) | | |
| Mean Slope^N^ | -13,369.489 | | 2,345.813 | 23,459.111 | | 20,851.965 | -11,318.381 | | |
|  | (18,579.721) | | (12,433.397) | (12,156.250) | | (11,612.366) | (6,349.982) | | |
| Km São Paulo | -7.370 | | -1.773 | -6.360 | | -6.963 | 2.400 | | |
|  | (13.965) | | (8.830) | (20.867) | | (15.905) | (4.844) | | |
| Km to River | -233.100 | | 63.322 | 1.649 | | -17.144 | -25.719 | | |
|  | (60.397)** | | (67.434) | (117.680) | | (139.618) | (72.181) | | |
| Open Area**_t-1_^N^** | 3.300 | | 2.374 | 2.408 | | 0.565 | -0.177 | | |
|  | (2.156) | | (2.010) | (1.873) | | (1.933) | (1.066) | | |
| Km to Road | 49.385 | | 22.328 | 13.340 | | 10.265 | 4.317 | | |
|  | (39.372) | | (37.495) | (53.504) | | (45.673) | (24.171) | | |
| Soil Type^N^ | Yes | | Yes | Yes | | Yes | Yes | | |
| Soil Texture^N^ | Yes | | Yes | Yes | | Yes | Yes | | |
| Constant | -7,131.150 | | 10,581.422 | 13,934.847 | | 16,571.464 | 5,464.878 | | |
|  | (21,619.354) | | (14,965.499) | (30,293.844) | | (24,775.364) | (8,021.285) | | |
| *R*^2^ | 0.29 | | 0.60 | 0.91 | | 0.84 | 0.71 | | |
| *N* | 139 | | 139 | 139 | | 139 | 139 | | |

* *p*<0.05; ** *p*<0.01

**Specification Set 4**

In the fourth set of specifications we estimate socioeconomic change over the full time interval of our dataset, as a function of change in agricultural area. For GDP, employment, and nighttime lights, we estimate change between 2001 and 2010 as a function of 2001 levels, agricultural area, and control variables. For agriculture and non-forest area (shown as *O*) change always refers to levels in 2001. Agricultural data is not available prior to 2001. We specify this model as equation (S4), where we specify changes in each socioeconomic variable as:

$\left( U_{i,2010}-U_{i,2001} \right)=\beta_{0}+\beta_{1}U_{i,2001}+\beta_{2}U_{i,2001}^{2}+\theta A_{i,2001}+\beta_{3}O_{i,2001}+\beta_{3}X_{i}+ \varepsilon_{i}$ (S4)

Population data, unfortunately, is not available for year 2001. Consequently, we estimate change in population as:

$\left( U_{i,2010}-U_{i,2000} \right)=\beta_{0}+\beta_{1}U_{i,2000}+\beta_{2}U_{i,2000}^{2}+\theta A_{i,2001}+\beta_{3}O_{i,2001}+\beta_{3}X_{i}+ \varepsilon_{i}$ (S5)

The full results from the fourth specification set were included in the main article body.

**Sensitivity Testing**

We recognize two possible sources of bias in our estimates. First, if urban growth is driving local agricultural production, then our estimates may be biased by revsese causality. Second, our definition and size of each agricultural neighborhood is based on an arbitrary threshold. We address these issues by (a) comparing the estimated values of theta that were obtained in specification sets 1-4 against an exogenous instrument for neighborhood agriculture and (b) by varying neighborhood threshold sizes.

*Reverse causality*

Past work on the broader impacts of agricultural growth on economic development has been complicated by the difficult task of parsing the impact of agriculture on other economic sectors from simultaneity and feedback effects. Urban growth, for example, could increase the demand for agricultural goods, which, in turn, could lead to an expansion of cropland areas in the city’s vicinity [8,9]. In this research, by focusing on an area defined by soybean production, a crop primarily destined for export, we are largely avoiding the possibility that urban growth is driving the region’s agricultural expansion. Nevertheless, we test our estimated coefficients for neighborhood agriculture against those obtained using an exogenous measure of *A* , which we denote as *Â*. If our estimates are biased by reverse feedbacks, or by agriculture being driven by local urban growth, then estimates of $\theta$ for *Â* should be less than those obtained by regressing on *A*.

We begin by estimating agriculture using a set of exogenous predictor variables available at the 5km^2^ grid cell level: most common soil characteristics, mean slope, mean elevation, and distance to the federal highways (the federal highways preceded Mato Grosso’s soybean sector), as well as a set of institutional variables: whether the majority of the area is protected and what biome covers the majority of the grid cell (biome determines what environmental laws apply). We then use the estimated coefficients to predict agricultural area for each grid cell. Note that our predictions change from year to year, as farmland expands into progressively less suitable areas. Finally, we calculate *Â* as the sum of *predicted* agriculture in each city’s neighborhood. We then substitute *Â* into Specification Sets S1-S3. We report the estimates for *θ*, using both *Â* and *A* in Tables S8. –S10.

If the estimates of *θ* obtained by regressing our dependent variables on *Â* are significantly less than those obtained by regressing eon *A*, then our results would suggest that our results are potentially biased by causal feedbacks from local urban growth on the regional agriculture sector. Our estimates, however, suggest that the opposite is occurring. When we substitute predicted agriculture for agriculture in specifications 1-2, our estimated coefficients are consistently larger than those estimated for actual agricultural area. We argue that this discrepancy is largely due to local farms’ reliance on nearby urban areas to provide services to the agricultural sector (e.g., the upstream and downstream linkages). The results obtained by regressing change in employment on *Â* are consistent with those obtained by regressing employment on *A.*

| **Table S8**  **Specification Set 1: IV Estimations.**  2001-2010, Panel data using fixed effects.  Specifications featuring actual and predicted agriculture. | | |  |  |
| --- | --- | --- | --- | --- |
| Specification | **Employment** | **GDP^+^** | **Employment** | **GDP^+^** |
| Agriculture^N^ | 2.18** (0.86) | 181** (58.6) | - | - |
| Predicted Agriculture^N^ | - | - | 3.97** (0.35) | 314** (28.32) |
| Non-Forest^N^ | 0.35 (0.58) | -16.8 (38.4) | -1.03 (0.38) | -103 (34.4) |
| Group Effects | Yes | Yes | Yes | Yes |
| Year Effects | Yes | Yes | Yes | Yes |
| (within) R² | 0.22 | 0.19 | 0.18 | 0.16 |
| Panel Years | 2010-2001 | 2010-2001 | 2010-2001 | 2010-2001 |
| N | 1251 | 1245 | 1250 | 1245 |
| **: p < .01 |  |  |  |  |

| **Table S9**  **Specification Set 2: IV estimations**  **Dependent Variable: Non-Agricultural Employment, 2001-2010**  **Estimates using both actual and predicted agricultural areas** | | | | | | | | |  |
| --- | --- | --- | --- | --- | --- | --- | --- | --- | --- |
|  | **2010** | **2009** | | **2008** | **2007** | | | **2006** |  |
| Agriculture_t-1_^N^ | 2.514 | | 2.300 | 2.140 | | 1.724 | 1.553 | | |
|  | (0.517)** | | (0.499)** | (0.471)** | | (0.637)** | (0.561)** | | |
| Predicted Agriculture_t-1_^N^ | 3.146 | | 2.809 | 2.820 | | 2.293 | 2.140 | | |
|  | (1.229)* | | (1.019)** | (0.892)** | | (0.943)* | (0.935)* | | |
|  | **2005** | | **2004** | **2003** | | **2002** |  | | |
| Agriculture_t-1_^N^ | 1.741 | | 1.700 | 1.218 | | 0.734 |  | | |
|  | (0.901) | | (0.795)* | (0.913) | | (0.573) |  | | |
| Predicted Agriculture_t-1_^N^ | 2.199 | | 2.405 | 1.983 | | 1.560 |  | | |
|  | (0.850)** | | (0.963)* | (01.260) | | (0.967) |  | | |
|  |  | |  |  | |  |  | | |
|  |  | |  |  | |  |  | | |

| **Table S10**  **Specification Set 3: IV estimations**  **Dependent Variable: Annual Change in Non-Agricultural Employment, 2001-2010**  **Coefficient s estimated using both actual and predicted agricultural areas** | | | | | | | | |  |
| --- | --- | --- | --- | --- | --- | --- | --- | --- | --- |
|  | **2010** | **2009** | | **2008** | **2007** | | | **2006** |  |
| Agriculture_t-1_^N^ | 0.211 | | 0.345 | 0.383 | | 0.156 | 0.083 | | |
|  | (0.073)** | | (0.037)** | (0.059)** | | (0.060)** | (0.090) | | |
| Predicted Agriculture_t-1_^N^ | 0.219 | | 0.377 | 0.442 | | 0.130 | -0.009 | | |
|  | (0.13) | | (0.088)** | (0.128)** | | (0.079) | (0.080) | | |
|  | **2005** | | **2004** | **2003** | | **2002** |  | | |
| Agriculture_t-1_^N^ | 0.040 | | 0.418 | 0.365 | | 0.189 |  | | |
|  | (0.113) | | (0.168)* | (0.179)* | | (0.103) |  | | |
| Predicted Agriculture_t-1_^N^ | 0.033 | | 5.62 | 0.487 | | 0.338 |  | | |
|  | (0.440) | | (0.238)* | (0.297) | | (0.150)* |  | | |
|  |  | |  |  | |  |  | | |
|  |  | |  |  | |  |  | | |

*Neighborhood Size*

The definition of neighborhoods was a key component of our analysis. However, our neighborhood threshold was based on an arbitrarily chosen sixty minute time threshold.

While we argue that sixty minutes represents a reasonable threshold for commuting from a farm to local urban area in Mato Grosso, we nevertheless seek to contextualize our estimates of *θ* using a sixty minute neighborhood against estimates of *θ* using a range of thresholds. We therefore test our models using a continuous array of specifications, varying the minute threshold from twenty to two hundred minutes, and compare the results.

We expected that *θ* would decrease as the minute threshold increased, in effect suggesting that the impact of agriculture on employment or nightlights would decrease as neighborhood area increases. In Figures S1a-S1b we estimate $\theta$ using the specifications from test two, set three, over a range of neighborhood thresholds. As we show in Figures S1a-S1b, $\theta$ declines to nearly zero at approximately 180 minutes travel time. However, while we expect that $\theta$ would decline, as agriculture relative to population or econoic growth increases, a significant question remains, namely how does the sum impact of agricultre on urban change vary with neighborhood size? We therefore simulate change in non-agricultural GDP and non-agricultural employment over this range of neighbrohood sizes. We present the results as Figures S2a and S2b.

(a) (b)

*Figures S1a-S1b. Estimated coefficients for* θ, *using a range of neighborhood sizes (in minutes, shown as x-axis). Sixty minutes corresponds to the estimates of* θ *shown for test t, in specification set three, in the principal manuscript.*

*Figures S2a-Sb. Estimated total new employment (S12a) and non-agricultural GDP (S12b) generated from agriculture, 2002-2010, over a range of neighborhood thresholds (in minutes, shown as x-axis). At sixty minutes the values correspond to values predicted in Figure 6 of the main manuscript.*
